# Supplementary material for: Proof of Concept of a 6-Month Person-Oriented Exercise Intervention ‘MultiPill-Exercise’ among Patients at Risk of or with Multiple Chronic Diseases: Results of a One-Group Pilot Trial
Source: Int J Environ Res Public Health. 2022 Aug 2;19(15):9469. doi: 10.3390/ijerph19159469 (PMC9368673; doi:10.3390/ijerph19159469)
Supplement: Supplementary file 1 [file ijerph-19-09469-s001.zip › Supplement S1 (Medication).pdf]

Table S1: Medication Survey. This questionnaire reported the medication of the last two weeks prior to the questionnaire.

**Medication**		**t0**	**t3**	**t6**
High Blood Pressure	Yes	24	20	16
	No	15	15	16
	Unknown / MV	0 / 0	0 / 4	0 / 7
High Blood Cholesterol	Yes	6	5	7
	No	24	26	23
	Unknown / MV	1 / 8	0 / 10	1 /8
Angina or Chest Pain	Yes	0	0	0
	No	30	30	27
	Unknown / MV	0 / 9	0 / 9	0 / 12
Control of Heart Rhythm	Yes	2	0	0
	No	27	29	27
	Unknown / MV	1 / 9	1 / 9	0 / 12
Heart Failure	Yes	0	0	0
	No	28	29	27
	Unknown / MV	1 / 10	1 / 9	0 / 12
Blood Thinning	Yes	4	4	3
	No	27	27	26
	Unknown / MV	0 / 8	0 / 8	0 / 10
Diabetes or High Blood Sugar	Yes	3	3	2
	No	28	28	25
	Unknown / MV	0 / 8	0 / 8	0 / 12
Stroke	Yes	0	0	0
	No	30	30	27
	Unknown / MV	0 / 9	0 / 9	0 / 12
Leg pain when walking	Yes	4	2	3
	No	26	28	25
	Unknown / MV	0 / 9	1 / 8	0 / 11
Aspirin, Alka-Seltzer, cold medicine or headache powder	Yes	10	11	10
	No	29	25	22
	Unknown / MV	0 / 0	0 / 3	0 / 7
Medication for Arthritis, fever, or muscle aches and pains, (or menstrual cramps)	Yes	3	8	6
	No	35	28	26
	Unknown / MV	1 / 0	0 / 3	0 / 7
Non-steroidal anti-inflammatory drugs	Yes	6	4	3
	No	32	31	28
	Unknown / MV	1 / 0	1 / 3	1 / 7

Medication survey according to ARIC Medication survey form [1]

1. Investigators, T.A. The Atherosclerosis Risk in Communities (ARIC) Study: design and objectives. The ARIC investigators. *Am J Epidemiol* **1989**, *129*, 687-702.
