# Supplementary material for: Proof of Concept of a 6-Month Person-Oriented Exercise Intervention ‘MultiPill-Exercise’ among Patients at Risk of or with Multiple Chronic Diseases: Results of a One-Group Pilot Trial
Source: Int J Environ Res Public Health. 2022 Aug 2;19(15):9469. doi: 10.3390/ijerph19159469 (PMC9368673; doi:10.3390/ijerph19159469)
Supplement: Supplementary file 1 [file ijerph-19-09469-s001.zip › Supplement S2 (PE Sensitivity analysis).pdf]

Sensitivity analysis without Multiple Imputation:

Complete Case Population (*n* = 29)

```
> summary(mod.1)
Model:
LD F1 Model

Call:
sports ~ Messzeitpunkte_num

Relative Treatment Effect (RTE):
                    RankMeans Nobs       RTE
Messzeitpunkte_num1  21.27586   29 0.2388030
Messzeitpunkte_num2  59.25862   29 0.6753864
Messzeitpunkte_num3  51.46552   29 0.5858105

Wald-Type Statistc (WTS):
                   Statistic df      p-value
Messzeitpunkte_num  113.2472  2 2.562602e-25

ANOVA-Type Statistc (ATS):
                   Statistic       df      p-value
Messzeitpunkte_num  37.35112 1.783427 2.174489e-15
```

#hypothesis H0 (T) of no time effect is tested with WTS and ATS --> overall significant so there is a time effect

Posthoc:

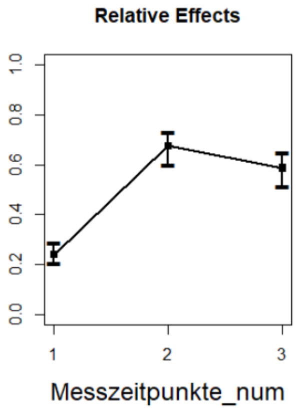


```
> #zeit 2 vs baseline 1
> p.out12.sensi
[1] 2.800301e-18
> #zeit 3 vs baseline 1
> p.out13.sensi
[1] 6.166443e-14
> #zeit 3 vs zeit 2
> p.out23.sensi
[1] 0.1695568
```

Bonferroni adjusted: t2 vs t3 corrected, p= 0.5086703
